# Impacts of PEGylation and Glycosylation on the Biological Properties of Host Defense Peptide IDR1018

**DOI:** 10.3390/pharmaceutics15051391

**Published:** 2023-05-01

**Authors:** Hashem Etayash, Fione Yip, Robert E. W. Hancock

**Affiliations:** Centre for Microbial Diseases and Immunity Research, Department of Microbiology and Immunology, University of British Columbia, 2259 Lower Mall Research Station, Vancouver, BC V6T 1Z4, Canada; etayashhashem@gmail.com (H.E.); mt3043mt@gmail.com (F.Y.)

**Keywords:** antimicrobial peptides, biofilm, anti-inflammatory, PEGylation, glycosylation

## Abstract

The multifunctional properties of host defense peptides (HDPs) make them promising drug candidates to tackle bacterial infections and tissue inflammation. However, these peptides tend to aggregate and can harm host cells at high doses, potentially limiting their clinical use and applications. In this study, we explored the influences of both pegylation and glycosylation on the biocompatibility and biological properties of HDPs, particularly the innate defense regulator IDR1018. Two peptide conjugates were designed by attaching either polyethylene glycol (PEG6) or a glucose moiety to the peptide towards the N-terminus. Significantly, both derivatives reduced the aggregation, hemolysis, and cytotoxicity of the parent peptide by orders of magnitude. In addition, while the pegylated conjugate, PEG6-IDR1018, retained an excellent immunomodulatory profile, similar to that observed for IDR1018 itself, the glycosylated conjugate, Glc-IDR1018, significantly outperformed the parent peptide in inducing anti-inflammatory mediators, MCP1 and IL-1RA and in suppressing the level of lipopolysaccharide-induced proinflammatory cytokine IL-1β. Conversely, the conjugates led to a partial reduction in antimicrobial and antibiofilm activity. These findings underline the impacts of both pegylation and glycosylation on the biological properties of the HDP IDR1018 and indicate the potential of glycosylation to enhance the design of highly effective immunomodulatory peptides.

## 1. Introduction

Bacterial infections are the second leading cause of death worldwide with an estimated 7.7 million deaths annually, of which 5 million are associated with antimicrobial resistance (AMR) to existing antibiotics [1]. They are a constant threat to global health systems and a continuing threat to human lives. Certainly, highly effective therapeutic interventions are needed more than ever to target bacterial infections and offset the rise in AMR. While various innovative approaches to discovering and developing new antibacterial agents have been proposed or undertaken such as the use of antibodies [2], aptamers [3], nucleic acid materials [4], nanoparticles of metal oxides [5,6,7], small synthetic chemical compounds [8,9], and others [10], antimicrobial host defense peptides (HDPs), which are part of the innate immune system, have been proven as a promising potential therapeutic strategy to confront bacterial infections and tackle the clinical threats of biofilm-forming drug-resistant strains due to the multiple advantages they offer [11]. HDPs are relatively small polymers (usually, 4–50 amino acid residues) that can be easily synthesized and modified at minimal costs. Unlike antibodies and small chemical compounds of nucleic acid-based drugs, antimicrobial HDPs often exhibit broad-spectrum activity against a wide range of bacteria species, including Gram-positive and Gram-negative pathogens, and usually, they tend to have a low propensity to develop antimicrobial resistance due to their multifaceted mechanism of actions [12,13]. These HDPs are also biodegradable, and tend to degrade easily; thus, they do not persist in the body and do not pose any undesirable side effects [13]. Furthermore, the ability of these antimicrobial peptides to disperse and eradicate mature bacterial biofilms and high-density bacterial infections both in vitro and in vivo gives them advantages over current antibiotics and other molecules as excellent antimicrobial and antibiofilm drug candidates [11]. Also, the ability of HDPs to act as anti-infective immune modulators with promising anti-inflammatory activity makes them clinically attractive candidates for multipurpose applications [14,15]. However, despite their advantages, as pharmaceutical products, HDPs endure several constraints that limit their ease of accessibility and use, including their systemic aggregation and toxic side effects typically at higher doses, in addition to their poor stability against blood-borne proteases [13,16]. While various approaches including, for instance, formulation strategies, sequence modifications and designing HDP mimetics have been explored to circumvent some of these shortcomings of HDPs, only limited successes for different peptides were achieved [13,17,18,19,20,21,22,23]. In this study, we decided to explore the special effects of both pegylation and glycosylation on the physiochemical properties as well as the biological activity properties of the HDP, IDR1018, which is a synthetic multifaceted peptide with immunomodulatory and antibiofilm activities [24,25]. Pegylation and glycosylation are modifications utilized in peptide/protein drug design and are often used to improve the stability and pharmacokinetic assets of potential drug candidates while reducing toxicities and potentially harmful side effects [26,27,28,29]. Pegylation is a process by which peptides are chemically conjugated to polyethylene glycol (PEG) in order to change their physicochemical or biological properties [30]. As PEG and its derivatives are inert, water-soluble, non-toxic and non-immunogenic, they are widely used to overcome limitations associated with biopharmaceutical products including water solubility issues, aggregations, toxicity against mammalian cells and immunogenicity [30]. Pegylation offers significant advantages for biopharmaceutical products, as attaching PEG improves proteolytic stability, helps mitigate the immunogenicity, increases resistance to bacterial-secreted enzymes, boosts blood circulation half-lives and enhances biodistribution as well as drug bioavailability [28,30]. Nevertheless, despite these advantages, pegylation is often associated with a partial or complete reduction in the antimicrobial activity of HDPs [30]. Glycosylation is a process by which a sugar moiety is chemically attached to biopharmaceutical molecules such as peptides, proteins, antibodies, etc., in order to change their physicochemical properties or produce better bioactive compounds [31]. As with pegylation, glycosylation can have a significant influence on the properties of HDPs, for example, modifying toxicities, resistance to proteolytic degradation, pharmacokinetics and dynamic properties [27,31,32]. However, as with pegylation, glycosylation does not always improve the antibacterial activity of HDPs since the attaching surges change the chemical structure, hydrophobicity and overall charge of the peptides, which can impact the insertion and interaction of peptides with bacterial membranes [31].

Indeed, the overall influence of pegylation and glycosylation on drug design is remarkable with many clinically accepted drugs being pegylated and glycosylated. Since little is known about the influence of both conjugations on the multifaceted properties of HDPs, especially the immunomodulatory functionality, we aimed in this study to look at their overall impacts by covalently modifying the antimicrobial HDP IDR1018 with short-chain PEG (PEG6) and a glucose moiety (N-acetyl glucosamine (GlcNAc)).

## 2. Materials and Methods

### 2.1. Peptide Synthesis, Pegylation, and Glycosylation

IDR1018 peptide (sequence, VRLIVAVRIWRR-NH_2_) was purchased from GenScript (Piscataway, NJ, USA) at >95% purity. The pegylated IDR1018 [PEG6; VRLIVAVRIWRR-NH_2_] as well as the glycosylated IDR1018 [Glc-IDR1018; T(GlcNAc)VRLIVAVRIWRR-NH_2_], were obtained at ≥95% purity from Biomatik LLC (Wilmington, DE, USA) (Table 1).

### 2.2. Aggregation Assay

Solutions of IDR1018 peptide and the conjugates were tested for aggregation in saline (0.9% NaCl), 5% dextrose, and 10% RPMI tissue culture medium at 1 mg mL^−1^. The solutions were placed in 96 well plates and sterile water was used as a negative control. The turbidity was determined for each sample as described in earlier reports [19,33]. Similarly, the conjugates were screened for aggregation in the presence of sodium salts of polyatomic anions (citrate or phosphate) at various strengths (0.1−1000 mM). The % of aggregates was assessed relative to the OD_600_ of sterile water.

**Figure 1:**
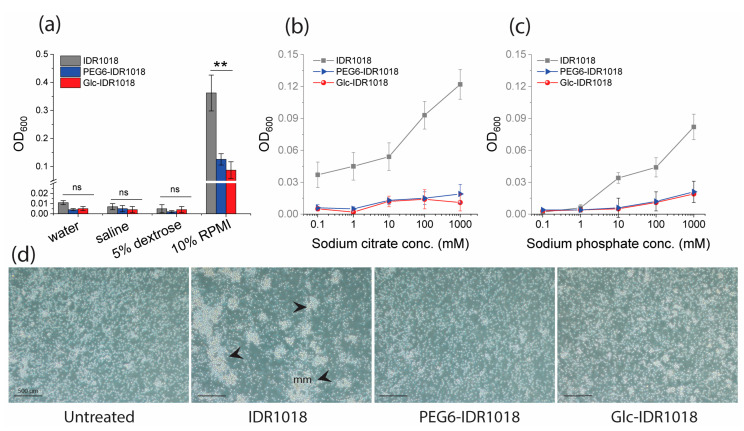
In vitro aggregation experiments of IDR1018 and its conjugates as tested in various solvents under different conditions. The aggregations of the peptides are indicated by the level of turbidity when dissolved in water, saline, 5% dextrose, or RPMI medium (**a**), or when dissolved in various concentrations of sodium citrate (**b**), and sodium phosphate (**c**). Microscopic images show PBMCs after treatment with IDR1018 or its conjugates, indicating aggregation by IDR1018 cf. the conjugates (**d**). Black arrows point to clusters that formed when PBMCs were treated with IDR1018 in a tissue culture medium. Data were described as means ± standard deviations, and significant values (**) were determined using the analysis of variance (ANOVA) test; ns in (**a**) indicates no significance. The scale bar is 500 µm.

Furthermore, isolated peripheral blood mononuclear cells (PBMCs) seeded in 96-well flat-bottom tissue culture plates (Corning Inc., Corning, NY, USA) at a concentration of 2 × 10^6^ in RMPI media were treated with the two conjugates at a final concentration of 32 μg mL^−1^, incubated for 4 h at 37 °C in 5% CO_2_, and visualized on a Nikon Eclipse TS100 microscope. Experiments were performed in triplicate in three independent experiments. Representative images of the microscopy are presented in Figure 1.

### 2.3. Hemolysis Test

Red blood cells (RBCs) were isolated and used to test the hemolytic activity of the peptide conjugates according to documented procedures [27,34] with some modifications. The blood samples were collected from healthy donors according to the University of British Columbia ethics certification and guidelines. Informed consent was obtained from all donors involved in the study. A 0.5% (*v*/*v*) RBC suspension in PBS was transferred into 96 well plates, where pre-diluted peptides series were incubated. The samples were placed at 37 °C for 1 h prior to reading the hemoglobin release at 414 nm and 546 nm. Triton X-100 (1% final concentration) was used as a positive control (100% hemolysis). The % hemolysis was then determined relative to the negative control (untreated samples).

### 2.4. Cytotoxicity and Immunomodulatory Assays

The cytotoxicity of the conjugates was assessed in vitro against PBMCs as described earlier using the lactate dehydrogenase (LDH)-based assay [35]. Blood samples were collected from healthy donors with ethics approval and PBMCs were isolated following our previously reported protocol [35]. The cells were resuspended in RPMI medium supplemented with 10% FBS and seeded in tissue culture-treated plates at a final density of 1 × 10^5^ cells well^−1^. Subsequently, the cells were treated with the conjugates or IDR1018 and incubated for 24 h at 37 °C in 5% CO_2_, to determine the LDH release. Triton X-100 (1% final concentration) was used as a toxic (positive) control.

To evaluate the capacity of the conjugates to modulate the immune system, suspended PBMCs (a final density of 1 × 10^5^ cells well^−1^) were treated with the conjugates at 8 μg mL^−1^ in the presence or absence of 20 ng mL^−1^ *P. aeruginosa* LPS, incubated for 24 h, centrifuged, and then the supernatants were collected to quantifying the levels of monocyte chemoattractant protein 1 (MCP-1), the tumor necrosis factor-alpha (TNFα), and interleukins (IL) IL-1β, IL-10, and the anti-inflammatory IL-1 receptor antagonist (IL-1RA) using specific ELISA assays (ELISA kits, eBioscience, Carlsbad, CA, USA). All experiments were conducted in triplicate in three independent experiments and average values were presented.

### 2.5. Minimal Inhibitory Concentration (MIC)

The MIC was conducted using the liquid broth inhibition assay as previously described [36]. MHB was used as a medium for bacterial growth, and bacteria (either MRSA SAP0017, *P. aeruginosa* PA14, *E. coli* ATTC489 or *A. baumannii* 5015) were suspended at ∼1 × 10^6^ CFU mL^−1^. The peptide conjugates were incubated with the bacterial cultures at various concentrations for ~18 h prior determination of bacterial growth optical density at 600 nm (OD_600_). The MIC was defined as the lowest concentration that led to no growth in the wells. All values are modal values of three independent experiments.

### 2.6. Biofilm Biomass and Biofilm Eradication Assays

The ability of the designed conjugates to decrease the biomass of bacterial biofilms was assessed in 96-well microtitre plates using the crystal violet staining assay as described previously [37]. A bacterial suspension of 1 × 10^7^ CFU mL^−1^ was used to establish biofilms prior to treatment with the peptides. Appropriate nutrient media for optimal biofilm formation were used, including TSB supplemented with 1% glucose for MRSA SAP0017, BM2 glucose for *P. aeruginosa* PA14, and LB media supplemented with 1% glucose for *E. coli* ATTC489 and *A. baumannii* 5015. In the crystal violet-based assay, untreated bacterial culture and sterile media were used as positive and negative controls, where the % of biofilm biomass was considered 100 and 0%, respectively. The % of biomass in the treated samples was determined relative to the positive and negative controls. A biofilm eradication assay [17] was also performed, for which the residual viable biofilm cell count (CFU mL^−1^) was determined. Experiments were conducted in triplicate, in three independent experiments, and average values were presented with ± standard deviations.

### 2.7. Statistical Analysis

GraphPad Prism 7.0 (GraphPad Software, La Jolla, CA, USA) and Origin Pro Software were used in the analysis. Whenever applicable, data were described as means ± standard deviations, and significant values were determined using the analysis of variance (ANOVA) test.

## 3. Results and Discussion

### 3.1. Peptide Conjugation Design

**Scheme 1:**
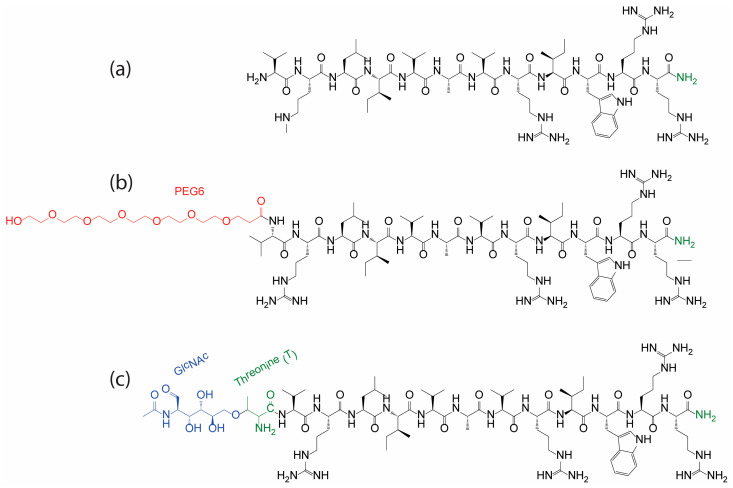
IDR1018 conjugates, (**a**) the antimicrobial HDP IDR1018, (**b**) the pegylated derivative PEG6-IDR1018, where a carboxylated PEG chain was attached to the N-terminal of the peptide and (**c**) the glycosylated derivative, Glc-IDR1018, where N-acetyl glucosamine (GlcNAc) was attached to the hydroxyl (-OH) group of the amino acid threonine (T).

The antimicrobial HDP IDR1018 was chosen for this study due to its promise as a therapeutic agent [38]. The well-known properties of IDR1018, including its ease and cost of synthesis as a linear peptide with 12 amino acid residues, as well as easily accessible sites for modifications, make it an excellent candidate for testing new modification approaches [17,19,38]. The original IDR1018 peptide sequence (Table 1) was modified by the addition of either PEG6 or a glucose moiety (GlcNAc) at the N-terminus to generate new derivatives, namely PEG6-IDR1018 and Glc-IDR1018, respectively. In PEG6-IDR1018, the PEG was attached to the amine terminus of the amino acid, valine, while in the glycosylation strategy, the GlcNAc was introduced into the peptide through O-linked glycosylation in an additional threonine residue. The resulting peptide conjugates are described in Table 1 while the chemical structures are shown in Scheme 1.

### 3.2. In Vitro Peptide(s) Aggregation

Aggregation is typically a limitation of HDPs since it not only impacts biological activity but also biocompatibility and immunogenicity [39,40]. In previous studies, the HDP IDR1018 was found to aggregate in various in vitro solvents as well as in vivo under certain conditions and as a consequence, a reduction in the immunomodulatory activity was observed under conditions that promote aggregation [19,33]. Here, we examined the aggregation tendency of peptide conjugates in multiple solutions, including water, saline (0.9% NaCl), 5% dextrose, and 10% RPMI tissue culture medium with 1% fetal bovine serum (FBS). While no aggregation (measured by turbidity) was observed in water, saline, or 5% dextrose for any sample, including IDR1018 (Figure 1a), a substantial increase in aggregation was detected in tissue culture medium for parent peptide IDR1018 but not for the pegylated and glycosylated derivatives.

When the peptides were prepared in sodium salts of abundant polyatomic anions, such as citrate and phosphate (Figure 1b,c), a substantial increase in turbidity was observed with IDR1018 at solute concentrations as low as 10 mM. In contrast, no turbidity increase was detected with PEG6-IDR1018 or Glc-IDR1018 at higher solute concentrations, suggesting a significant reduction in the salt-induced peptide aggregation. When incubated with PBMCs in tissue culture, neither peptide conjugate, PEG6-IDR1018, and Glc-IDR1018 showed any obvious sign of aggregation, in strong contrast to IDR1018, which showed amorphous aggregates (Figure 1d). These results, indeed, were consistent with earlier reports, that demonstrated the utility of pegylation and glycosylation in increasing solubility and diminishing aggregation for other antimicrobial peptides [31,41].

### 3.3. Hemolysis, Cytotoxicity and Immunomodulatory Activity

**Figure 2:**
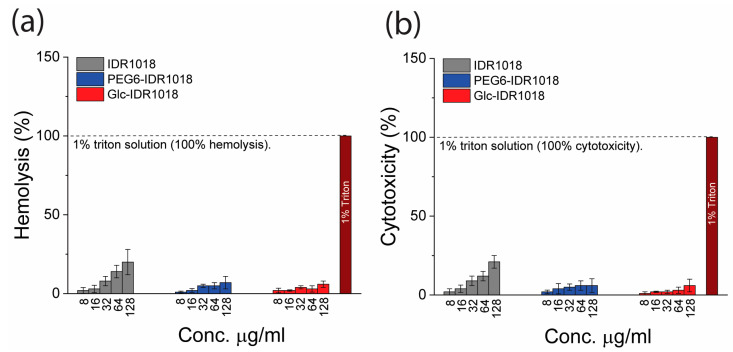
Hemolysis and Cytotoxicity of the peptide conjugates vs. IDR1018 itself. (**a**) displays the hemolysis analysis against RBCs while (**b**) depicts the cytotoxicity against PBMCs. Experiments were conducted in triplicate in three independent experiments and mean values ± standard deviation are presented.

Compared to the parent IDR1018 peptide, which appeared to be hemolytic against RBCs as well as toxic to PBMCs at high doses (up to 20% at 128 µg mL^−1^), neither of the conjugated derivatives exhibited any hemolysis or cytotoxicity at the highest concentration tested (Figure 2a,b). Consistent with other previous studies for other peptides [30,31,42,43], pegylation and glycosylation worked well in reducing HDP toxicity.

**Figure 3:**
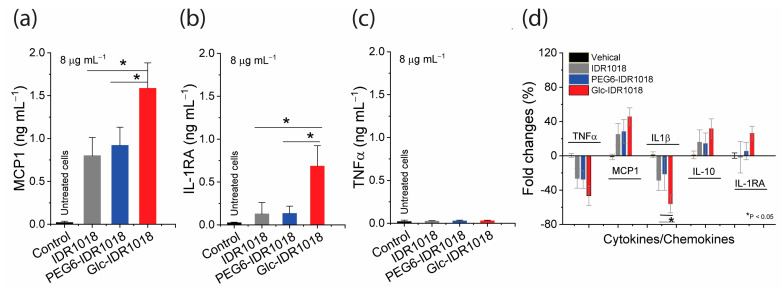
Enhanced effect of glycosylation on the immunomodulatory activities of IDR1018-derived peptides. (**a**–**c**) The effect of the peptides at 8 μg mL^−1^ in inducing chemokine MCP1 and cytokines IL-1RA and TNFα production by PBMCs in the absence of LPS stimulation. (**d**) the effect of peptides on the immune mediator expression when co-treated with 20 ng mL^−1^ LPS (% of fold changes relative to LPS-stimulated cells). Data are presented as mean ± standard deviation from three independent experiments (* represents *p* < 0.05, based on ANOVA analysis with Tukey’s correction for multiple testing).

The capacity of conjugated peptide derivatives to modulate the innate immune system was tested by assessing the induction and suppression of chemokines and cytokines from human PBMCs. These tested modulators including MCP1, TNFα, IL-1β, IL-1RA and IL-10, were selected based on previous studies where IDR1018 demonstrated an excellent ability to modulate them in vitro and in vivo [17,19,24,38,44,45]. In addition, these immunity modulators are involved in multiple systemic mechanisms essential to suppress inflammations and indirectly defend against bacterial infections. For instance, MCP-1 attracts macrophages and enhances the recruitment of monocytes at the sites of infections, injuries and inflammations [46], while the anti-inflammatory cytokines, IL-1RA and IL-10 act as natural inhibitors of the harmful effect of pro-inflammatory immune mediators such as IL-1α and IL-1β [47]. Interestingly, in the absence of an LPS stimulant, PEG6-IDR1018 maintained a similar ability to modulate chemokine and cytokine expression to that observed for unmodified IDR1018. However, Glc-IDR1018 exhibited a significant increase in the expression of the immune mediators relative to unmodified IDR1018. Thus Glc-IDR1018 boosted the level of MCP1 and IL-1RA by 2–5 fold,1.5 ng mL^−1^ and 0.8 ng mL^−1^, respectively, compared to only 0.7 and 0.15 ng mL^−1^ induced by the unmodified IDR1018 (Figure 3a,b). These peptides did not induce TNFα (Figure 3c).

When stimulated with LPS which amplified the secretion of immune mediators, substantial reductions in the proinflammatory TNFα were detected due to peptide action, with no significant difference between all three peptides. However, the expression of IL-1β was significantly suppressed when cells were treated with Glc-IDR1018 cf. the other peptides (Figure 3d). Moreover, remarkable peptide-induced increases in MCP1, IL-1RA and IL-10 induction levels were also detected with substantially higher expression values associated with Glc-IDR1018 treatment. In agreement with previous reports, the results indicated that pegylation had no marked effect on the immunomodulatory properties of the HDP IDR1018 [43,48]. However, this was the first report we have observed indicating that glycosylation markedly improves the immunomodulatory properties of HDPs. While the mechanistic effects of glycosylation on the immunomodulatory properties of IDR1018 as well as the underlying biophysical processes remain to be elucidated, the overall results showed exciting data with excellent outcomes for this peptide conjugate in modulating the immune system with efficacy exceeding that for previously reported HDPs [15,19].

### 3.4. Antimicrobial Activity

The two peptide conjugates (Table 1) were intended to mimic and/or enhance the activity of the parent IDR1018 while improving biocompatibility and physicochemical properties. Despite an enhancement or retention of immunomodulatory activities, there was a 2 to 8-fold reduction in antimicrobial activity (i.e., higher MIC values) when compared to the parent IDR1018 (Table 2).

The MICs of the PEG6-IDR1018 ranged between 32–64 µg mL^−1^, while the MICs of the Glc-IDR1018 ranged between 64–128 µg mL^−1^ based on the strains tested. According to the literature, [26,29,43,49,50], such decreases in antimicrobial activity are not uncommon for PEGylated and glycosylated peptides. The activity of the antimicrobial peptide Nisin, for instance, was reduced by conjugation with polyethylene glycol [50]. Likewise, the activity of other antimicrobial HDPs, such as α defensine 1 [51], tachyplestin I [52], magainin 2 [49], aurein 2.2 [53] and others [26], all exhibited reduced antimicrobial activity when pegylated. It is worth noting that previous results for pegylation and glycosylation appear to depend on the type of ligation, the PEG or glycan composition and structure, the nature of the PEG or the sugar-peptide bond and their lengths, and the overall structural conformation of the generated molecules [54,55]. In a study by Falciani C et al., for instance, pegylation at the C-terminus of the antimicrobial peptide M33 was shown to improve the stability of the peptide against *Pseudomonas aeruginosa* elastase [56]. While on the contrary, conjugation at the N-terminus of HDPs, LL-37 and cecropin A, derivatives (PEG-CaLL), showed to improve the antimicrobial activity against various bacterial strains, including *B. anthracis* (including vegetative and spore forms), *Escherichia coli* and *Staphylococcus aureus* when compared to the LL-37 peptide [57]. By analogy, in a study by Talat et al., the activity of the glycosylated peptides was shown to be impacted by the stereochemistry of attached sugars [58]. The study also showed that β-linked sugars induce more flexible and conformationally unstrained conjugates in contrast to the α-linked counterparts which cause more rigid and highly stable conjugates [58]. Although the N and C termini of peptides tend to show conformational flexibility, further studies would be required to investigate if these conjugations impacted the overall HDP IDR1018 chemical structure including its secondary structure, in order to understand the potential reasons behind the reduced activity. It would also be worthwhile investigating the impact of these conjugations on other antimicrobial HDPs, including IDR1018 analogs.

### 3.5. Antibiofilm Activity

**Figure 4:**
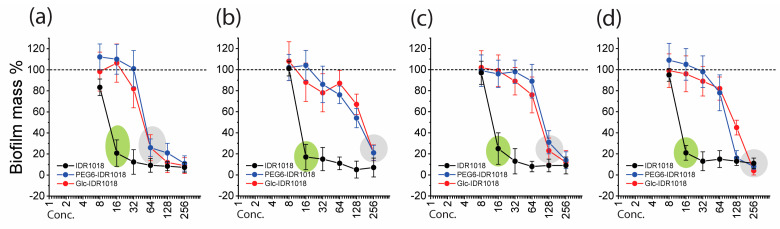
Effect of the peptide conjugates in reducing bacterial biofilm mass as assessed by the crystal violet staining assay. (**a**–**d**) show the antibiofilm activity against *S. aureus* MRSA0017, *P. aeruginosa* PA14, *E. coli* ATTC489, and *A. baumannii* 5015, respectively. The % of biofilm mass reduction was determined relative to untreated samples. The data represents the mean values ± standard deviation. All experiments were conducted three times. The green and grey shades indicate relative MBECs.

Effects on bacterial biomass were first tested using a crystal violet staining assay. While none of the peptides exhibited complete eradication of the biomass staining in the wells at the MIC values, the results showed a reduced ability of the designated conjugates to reduce the adhered biofilm mass by >90%, at much higher concentrations compared to the original IDR1018 (Figure 4). The best antibiofilm activity of the conjugates was observed against *S. aureus* MRSA0017, where ≥90% of the biomass reduction was observed at 64 µg mL^−1^ for both PEG6-IDR1018 and Glc-IDR1018 peptides. The results were supported by determining the viable biofilm cell counts (CFU) following biofilm treatments in the biofilm eradication assay (where biofilms were pre-formed), indicating comparable outcomes to the biomass dispersal results (Table 3). The peptide conjugates exhibited a 3 to 16-fold reduction in the antibiofilm activity (i.e., higher minimal biofilm eradication concentration values or MBEC = 64–256 µg mL^−1^) compared to the unmodified IDR1018 (MBEC = 16 µg mL^−1^) for all tested strains. Thus, in agreement with the MIC results, the pegylation and glycosylation led to maintenance but a weakening of the antibiofilm properties of the IDR1018. It is clear from these results that pegylation and glycosylation modifications did not generate more efficacious antimicrobial or antibiofilm peptides and that their influences on the HDPs’ antimicrobial and antibiofilm activity seemed peptide-specific.

## 4. Conclusions

Two conjugated derivatives of the antimicrobial HDP IDR1018 were designed based on N-terminal pegylation and glycosylation, in order to improve the biocompatibility and biological properties of the original peptide sequence. While additional studies remain to be explored, such as using different sugar moieties, different lengths of PEG chains, and different ligation approaches, the current conjugates significantly reduced the aggregation, hemolysis, and cytotoxicity of the parent peptide. Moreover, while pegylation conserved the immunomodulatory properties of the peptide, glycosylation generated a superior immunomodulating conjugate with a strong ability to stimulate the release of chemokine MCP-1 and the anti-inflammatory cytokine IL-1RA while suppressing the LPS-induced production of proinflammatory cytokines TNFα and IL-1β, suggesting a novel anti-inflammatory drug candidate. Consistent with several previously reported studies, both conjugations led to partial reductions in antimicrobial as well as antibiofilm activity cf. the original IDR1018. Overall, the study reported here clearly demonstrates the usefulness of these conjugation approaches in optimizing the physicochemical properties of therapeutic peptides and highlight a new potential opportunity for enhancing immunomodulatory HDPs through glycosylation.

## Figures and Tables

**Table 1:** IDR1018 and its conjugated derivatives, HPLC, and mass spectra data.

Peptide and Derivatives	Sequence	HPLC (Ret. Time (min))	MW (Calc.)	MW (Obs.)	Purity (%)
IDR1018	VRLIVAVRIWRR-NH_2_	9.1	1535.8	1535.8	>95.0
PEG6-IDR1018	PEG6-VRLIVAVRIWRR-NH_2_	17.9	1872.3	1872.6	>95.0
Glc-IDR1018	T(GlcNAc)VRLIVAVRIWRR-NH_2_	14.3	1841.4	1841.3	>92.0

HPLC, high-performance liquid chromatography; Ret. Time, retention time in minutes; MW, molecular weight; Calc., calculated; Obs., observed.

**Table 2:** Activities of the peptide conjugates vs. planktonic bacteria.

Bacteria	MIC (μg mL^−1^)		
	IDR1018	PEG6-IDR1018	Glc-IDR1018
*S. aureus* MRSA0017	16	32	64
*P. aeruginosa* PA14	16	64	128
*E. coli* ATTC489	16	64	64
*A. baumannii* 5015	16	64	64

**Table 3:** Activities of the peptide conjugates vs. preformed bacterial biofilms (minimal biofilm eradication concentrations, MBECs).

Bacteria	MBEC (μg mL^−1^)		
	IDR1018	PEG6-IDR1018	Glc-IDR1018
*S. aureus* MRSA0017	16	64	64
*P. aeruginosa* PA14	16	256	256
*E. coli* ATTC489	16	128	128
*A. baumannii* 5015	16	128	256

## Data Availability

Not applicable.

## References

[1] Ikuta K.S., Swetschinski L.R., Aguilar G.R., Sharara F., Mestrovic T., Gray A.P., Weaver N.D., Wool E.E., Han C., Hayoon A.G. (2022). Global mortality associated with 33 bacterial pathogens in 2019: A systematic analysis for the global burden of disease study 2019. Lancet.

[2] El-Kafrawy S.A., Abbas A.T., Oelkrug C., Tahoon M., Ezzat S., Zumla A., Azhar E.I. (2023). IgY antibodies: The promising potential to overcome antibiotic resistance. Front. Immunol..

[3] Li W. (2020). Prospective Application of aptamer-based assays and therapeutics in bloodstream infections. Mini Rev. Med. Chem..

[4] Sun Y., Meng L., Zhang Y., Zhao D., Lin Y. (2021). The application of nucleic acids and nucleic acid materials in antimicrobial research. Curr. Stem Cell Res. Ther..

[5] Pino P., Bosco F., Mollea C., Onida B. (2023). Antimicrobial nano-zinc oxide biocomposites for wound healing applications: A Review. Pharmaceutics.

[6] Akshaya S., Rowlo P.K., Dukle A., Nathanael A.J. (2022). Antibacterial coatings for titanium implants: Recent trends and future perspectives. Antibiotics.

[7] Sun C., Wang X., Dai J., Ju Y. (2022). Metal and metal oxide nanomaterials for fighting planktonic bacteria and biofilms: A review emphasizing on mechanistic aspects. Int. J. Mol. Sci..

[8] Zha G.F., Preetham H.D., Rangappa S., Sharath Kumar K.S., Girish Y.R., Rakesh K.P., Ashrafizadeh M., Zarrabi A., Rangappa K.S. (2021). Benzimidazole analogues as efficient arsenals in war against methicillin-resistance *Staphylococcus aureus* (MRSA) and its SAR studies. Bioorg. Chem..

[9] Mousavifar L., Roy R. (2021). Recent development in the design of small ‘drug-like’ and nanoscale glycomimetics against *Escherichia coli* infections. Drug Discov. Today.

[10] Hemmati F., Rezaee M.A., Ebrahimzadeh S., Yousefi L., Nouri R., Kafil H.S., Gholizadeh P. (2021). Novel strategies to combat bacterial biofilms. Mol. Biotechnol..

[11] Hancock R.E.W., Alford M.A., Haney E.F. (2021). Antibiofilm activity of host defence peptides: Complexity provides opportunities. Nat. Rev. Microbiol..

[12] Magana M., Pushpanathan M., Santos A.L., Leanse L., Fernandez M., Ioannidis A., Giulianotti M.A., Apidianakis Y., Bradfute S., Ferguson A.L. (2020). The value of antimicrobial peptides in the age of resistance. Lancet Infect. Dis..

[13] Etayash H., Hancock R.E.W. (2021). Host defense peptide-mimicking polymers and polymeric-brush-tethered host defense peptides: Recent developments, limitations, and potential success. Pharmaceutics.

[14] Kim J., Cho B.H., Jang Y.S. (2023). Understanding the roles of host defense peptides in immune modulation: From antimicrobial action to potential as adjuvants. J. Microbiol. Biotechnol..

[15] Hancock R.E.W., Haney E.F., Gill E.E. (2016). The immunology of host defence peptides: Beyond antimicrobial activity. Nat. Rev. Immunol..

[16] Ting D.S.J., Beuerman R.W., Dua H.S., Lakshminarayanan R., Mohammed I. (2020). Strategies in translating the therapeutic potentials of host defense peptides. Front. Immunol..

[17] Etayash H., Alford M., Akhoundsadegh N., Drayton M., Straus S.K., Hancock R.E.W. (2021). Multifunctional antibiotic-host defense peptide conjugate kills bacteria, eradicates biofilms, and modulates the innate immune response. J. Med. Chem..

[18] Etayash H., Qian Y., Pletzer D., Zhang Q., Xie J., Cui R., Dai C., Ma P., Qi F., Liu R. (2020). Host defense peptide-mimicking amphiphilic β-peptide polymer (Bu:DM) exhibiting anti-biofilm, immunomodulatory, and in vivo anti-infective activity. J. Med. Chem..

[19] Etayash H., Pletzer D., Kumar P., Straus S.K., Hancock R.E.W. (2020). Cyclic derivative of host-defense peptide IDR-1018 improves proteolytic stability, suppresses inflammation, and enhances in vivo activity. J. Med. Chem..

[20] Gonçalves da Costa Sousa M., Conceição de Almeida G., Martins Mota D.C., Andrade da Costa R., Dias S.C., Limberger S.N., Ko F., Lin L.T., Haney E.F., Etayash H. (2022). Antibiofilm and immunomodulatory resorbable nanofibrous filing for dental pulp regenerative procedures. Bioact. Mater..

[21] Thapa R.K., Diep D.B., Tønnesen H.H. (2020). Topical antimicrobial peptide formulations for wound healing: Current developments and future prospects. Acta Biomater..

[22] Gaglio S.C., Jabalera Y., Montalbán-López M., Millán-Placer A.C., Lázaro-Callejón M., Maqueda M., Carrasco-Jimenez M.P., Laso A., Aínsa J.A., Iglesias G.R. (2022). Embedding biomimetic magnetic nanoparticles coupled with peptide AS-48 into PLGA to treat intracellular pathogens. Pharmaceutics.

[23] van Gent M.E., Ali M., Nibbering P.H., Kłodzińska S.N. (2021). Current advances in lipid and polymeric antimicrobial peptide delivery systems and coatings for the prevention and treatment of bacterial infections. Pharmaceutics.

[24] Wieczorek M., Jenssen H., Kindrachuk J., Scott W.R., Elliott M., Hilpert K., Cheng J.T., Hancock R.E.W., Straus S.K. (2010). Structural studies of a peptide with immune modulating and direct antimicrobial activity. Chem. Biol..

[25] Hilchie A.L., Wuerth K., Hancock R.E.W. (2013). Immune modulation by multifaceted cationic host defense (antimicrobial) peptides. Nat. Chem. Biol..

[26] Rezende S.B., Oshiro K.G.N., Júnior N.G.O., Franco O.L., Cardoso M.H. (2021). Advances on chemically modified antimicrobial peptides for generating peptide antibiotics. Chem. Commun..

[27] Grimsey E., Collis D.W.P., Mikut R., Hilpert K. (2020). The effect of lipidation and glycosylation on short cationic antimicrobial peptides. Biochim. Biophys. Acta Biomembr..

[28] Manteghi R., Pallagi E., Olajos G., Csóka I. (2020). Pegylation and formulation strategy of anti-microbial peptide (AMP) according to the quality by design approach. Eur. J. Pharm. Sci..

[29] Bednarska N.G., Wren B.W., Willcocks S.J. (2017). The importance of the glycosylation of antimicrobial peptides: Natural and synthetic approaches. Drug Discov. Today.

[30] Harris J.M., Chess R.B. (2003). Effect of pegylation on pharmaceuticals. Nat. Rev. Drug Discov..

[31] Bellavita R., Braccia S., Galdiero S., Falanga A. (2023). Glycosylation and lipidation strategies: Approaches for improving antimicrobial peptide efficacy. Pharmaceuticals.

[32] Moradi S.V., Hussein W.M., Varamini P., Simerska P., Toth I. (2016). Glycosylation, an effective synthetic strategy to improve the bioavailability of therapeutic peptides. Chem. Sci..

[33] Haney E.F., Wu B.C., Lee K., Hilchie A.L., Hancock R.E.W. (2017). Aggregation and its influence on the immunomodulatory activity of synthetic innate defense regulator peptides. Cell Chem. Biol..

[34] Rodríguez A., Villegas E., Montoya-Rosales A., Rivas-Santiago B., Corzo G. (2014). Characterization of antibacterial and hemolytic activity of synthetic pandinin 2 variants and their inhibition against *Mycobacterium tuberculosis*. PLoS ONE.

[35] Etayash H., Haney E.F., Hancock R.E.W. (2021). Assessing biofilm inhibition and immunomodulatory activity of small amounts of synthetic host defense peptides synthesized using SPOT-array technology. Nat. Protoc..

[36] Wiegand I., Hilpert K., Hancock R.E.W. (2008). Agar and broth dilution methods to determine the minimal inhibitory concentration (MIC) of antimicrobial substances. Nat. Protoc..

[37] Haney E.F., Trimble M.J., Hancock R.E.W. (2021). Microtiter plate assays to assess antibiofilm activity against bacteria. Nat. Protoc..

[38] Mansour S.C., de la Fuente-Núñez C., Hancock R.E.W. (2015). Peptide IDR-1018: Modulating the immune system and targeting bacterial biofilms to treat antibiotic-resistant bacterial infections. J. Pept. Sci..

[39] Zou R., Zhu X., Tu Y., Wu J., Landry M.P. (2018). Activity of antimicrobial peptide aggregates decreases with increased cell membrane embedding free energy cost. Biochemistry.

[40] Zai Y., Xi X., Ye Z., Ma C., Zhou M., Chen X., Siu S.W.I., Chen T., Wang L., Kwok H.F. (2021). Aggregation and its influence on the bioactivities of a novel antimicrobial peptide, Temporin-PF, and its analogues. Int. J. Mol. Sci..

[41] Kumar P., Pletzer D., Haney E.F., Rahanjam N., Cheng J.T.J., Yue M., Aljehani W., Hancock R.E.W., Kizhakkedathu J.N., Straus S.K. (2019). Aurein-derived antimicrobial peptides formulated with pegylated phospholipid micelles to target methicillin-resistant *Staphylococcus aureus* skin infections. ACS Infect. Dis..

[42] Moreira Brito J.C., Carvalho L.R., Neves de Souza A., Carneiro G., Magalhães P.P., Farias L.M., Guimarães N.R., Verly R.M., Resende J.M., Elena de Lima M. (2022). PEGylation of the antimicrobial peptide LyeTx I-b maintains structure-related biological properties and improves selectivity. Front. Mol. Biosci..

[43] Singh S., Papareddy P., Mörgelin M., Schmidtchen A., Malmsten M. (2014). Effects of PEGylation on membrane and lipopolysaccharide interactions of host defense peptides. Biomacromolecules.

[44] Steinstraesser L., Hirsch T., Schulte M., Kueckelhaus M., Jacobsen F., Mersch E.A., Stricker I., Afacan N., Jenssen H., Hancock R.E.W. (2012). Innate defense regulator peptide 1018 in wound healing and wound infection. PLoS ONE.

[45] Rivas-Santiago B., Castañeda-Delgado J.E., Rivas Santiago C.E., Waldbrook M., González-Curiel I., León-Contreras J.C., Enciso-Moreno J.A., del Villar V., Mendez-Ramos J., Hancock R.E.W. (2013). Ability of innate defence regulator peptides IDR-1002, IDR-HH2 and IDR-1018 to protect against *Mycobacterium tuberculosis* infections in animal models. PLoS ONE.

[46] Deshmane S.L., Kremlev S., Amini S., Sawaya B.E. (2009). Monocyte chemoattractant protein-1 (MCP-1): An overview. J. Interf. Cytokine Res..

[47] Yazdi A.S., Ghoreschi K. (2016). The interleukin-1 family. Adv. Exp. Med. Biol..

[48] Haney E.F., Wuerth K.C., Rahanjam N., Safaei Nikouei N., Ghassemi A., Alizadeh Noghani M., Boey A., Hancock R.E.W. (2019). Identification of an IDR peptide formulation candidate that prevents peptide aggregation and retains immunomodulatory activity. Pept. Sci..

[49] Imura Y., Nishida M., Matsuzaki K. (2007). Action mechanism of PEGylated magainin 2 analogue peptide. Biochim. Biophys. Acta.

[50] Guiotto A., Pozzobon M., Canevari M., Manganelli R., Scarin M., Veronese F.M. (2003). PEGylation of the antimicrobial peptide nisin A: Problems and perspectives. Farmaco.

[51] Wu Z., Li X., Ericksen B., de Leeuw E., Zou G., Zeng P., Xie C., Li C., Lubkowski J., Lu W.Y. (2007). Impact of pro segments on the folding and function of human neutrophil alpha-defensins. J. Mol. Biol..

[52] Imura Y., Nishida M., Ogawa Y., Takakura Y., Matsuzaki K. (2007). Action mechanism of tachyplesin I and effects of PEGylation. Biochim. Biophys. Acta.

[53] Kumar P., Takayesu A., Abbasi U., Kalathottukaren M.T., Abbina S., Kizhakkedathu J.N., Straus S.K. (2017). Antimicrobial peptide-polymer conjugates with high activity: Influence of polymer molecular weight and peptide sequence on antimicrobial activity, proteolysis, and biocompatibility. ACS Appl. Mater. Interfaces.

[54] Talat S., Thiruvikraman M., Kumari S., Kaur K.J. (2011). Glycosylated analogs of formaecin I and drosocin exhibit differential pattern of antibacterial activity. Glycoconj. J..

[55] Li T., Yang N., Teng D., Mao R., Hao Y., Wang X., Wang J. (2022). C-terminal mini-PEGylation of a marine peptide N6 had potent antibacterial and anti-inflammatory properties against *Escherichia coli* and *Salmonella* strains in vitro and in vivo. BMC Microbiol..

[56] Falciani C., Lozzi L., Scali S., Brunetti J., Bracci L., Pini A. (2014). Site-specific pegylation of an antimicrobial peptide increases resistance to Pseudomonas aeruginosa elastase. Amino Acids.

[57] Morris C.J., Beck K., Fox M.A., Ulaeto D., Clark G.C., Gumbleton M. (2012). Pegylation of antimicrobial peptides maintains the active peptide conformation, model membrane interactions, and antimicrobial activity while improving lung tissue biocompatibility following airway delivery. Antimicrob. Agents Chemother..

[58] Coltart D.M., Royyuru A.K., Williams L.J., Glunz P.W., Sames D., Kuduk S.D., Schwarz J.B., Chen X.T., Danishefsky S.J., Live D.H. (2002). Principles of mucin architecture: Structural studies on synthetic glycopeptides bearing clustered mono-, di-, tri-, and hexasaccharide glycodomains. J. Am. Chem. Soc..

